# Dual Crosslinked Methacrylated Alginate Hydrogel Micron Fibers and Tissue Constructs for Cell Biology

**DOI:** 10.3390/md17100557

**Published:** 2019-09-28

**Authors:** Yingjun Gao, Xiangyu Jin

**Affiliations:** Key Laboratory of Textile Science and Technology of the Ministry of Education, College of Textiles, Donghua University, Shanghai 201620, China; gaoyingj@hotmail.com

**Keywords:** alginate, methacrylation, microfluidic fabrication, dual crosslinking, cell-encapsulated, biological microfibers assemblies, marine biomaterial

## Abstract

As an important natural polysaccharide biomaterial from marine organisms, alginate and its derivatives have shown great potential in the fabrication of biomedical materials such as tissue engineering, cell biology, drug delivery, and pharmaceuticals due to their excellent biological activity and controllable physicochemical properties. Ionic crosslinking is the most common method used in the preparation of alginate-based biomaterials, but ionic crosslinked alginate hydrogels are prone to decompose in physiological solution, which hinders their applications in biomedical fields. In this study, dual crosslinked alginate hydrogel microfibers were prepared for the first time. The ionic crosslinked methacrylated alginate (Alg-MA) hydrogel microfibers fabricated by Microfluidic Fabrication (MFF) system were exposed to ultraviolet (UV) light and covalent crosslink between methacrylate groups avoided the fracture of dual crosslinked macromolecular chains in organizational environment. The chemical structures, swelling ratio, mechanical performance, and stability were investigated. Cell-encapsulated dual crosslinked Alg-MA hydrogel microfibers were fabricated to explore the application in tissue engineering for the first time. The hydrogel microfibers provided an excellent 3D distribution and growth conditions for cells. Cell-encapsulated Alg-MA microfibers scaffolds with functional 3D tissue structures were developed which possessed great potential in the production of next-generation scaffolds for tissue engineering and regenerative medicine.

## 1. Introduction

Hydrogels possess excellent biocompatibility and unique moisture retention properties, which can provide conditions similar to the cell growth environment in vivo for cells embedded in it [1,2,3]. The fabrication of cell-laden or cell-embedded hydrogel biomaterials including microfibers, scaffold, microbeads, nano- or micro- capsules, etc., has become hot research topic in the field of biomaterials and these hydrogels show excellent performance as fundamental components in the fields of tissue engineering, cell biology, and tissue regeneration [4,5,6,7,8,9,10,11]. Microfluidic fabrication (MFF) technologies are known as a promising platform to provide an accurate control of the fibers which is useful in the fabrication of micro- and nano-scale hydrogel biomaterials. The MFF system contains two or more independent microfluidic channels, which contain different solutions. These solutions flow coaxially and separate from each other. When the solutions flow out of the channels, microfibers are fabricated at the interface of the solutions by using ion crosslinking or photopolymerization processes. Compared with other microfiber fabrication techniques, the MFF system has the advantage of using simple equipment to fabricate microfibers with diverse shapes and sizes. Hydrogel microfibers fabricated by MFF technologies have shown significant potential as scaffolds for tissue engineering and cell biology. Cell-laden hydrogel microfibers are promising cell carriers for tissue engineering and regenerative medicine, which can be easily prepared by using MFF technologies by adding specific cells into the solution before or during spinning [12,13,14,15,16,17]. Cell-laden hydrogel microfibers with various structures (e.g., tubular, porous, grooves, flat, and hollow) can be obtained by changing the spinning solution, experimental parameters (solution flow rate and mode, crosslinking time, etc.) and the microfluidic platforms (rectangular, cylindrical, flat, and pattern) to meet the requirements of specific tissue engineering or cell biology applications [17,18,19,20,21].

Sodium alginates are naturally derived marine polysaccharides and composed of two uronic acids, (1-4)-linked α-L-guluronic acid (G units) and β-D-manuronic acid (M units) [22]. Sodium alginate possesses a unique ability to chelate with multivalent cations, such as the G-G blocks can chelate with calcium ions in solution and form insoluble calcium alginate with “egg-box” molecular structure [23,24]. Therefore, ionic crosslinked alginate hydrogels with various structures can be prepared easily. Alginate hydrogels have favourable biological properties, such as biocompatibility, non-antigenicity and biodegradability and are extensively used in wound healing, drug delivery, tissue engineering, cell encapsulation, and cell transplantation [25,26,27,28,29]. The application of calcium ionic crosslinked microfibers in cell carriers are researched, and demonstrated that cells remain viable in fibers [30]. However, the stability of structure and properties of calcium ionic crosslinked in physiological solution is poor. In physiological solutions, calcium ions in calcium alginate can be exchanged by other ions and water-soluble macromolecules, such as sodium alginate. So that the structure and mechanical properties of calcium ionic crosslinked hydrogels can be destroyed and eventually lose its stable 3D structure [22,31,32]. This has been hampering the applications of calcium alginate hydrogels in biomedical materials.

The mechanical properties and structural stability of the photo-crosslinked alginate hydrogels are much better than that of the calcium ionic crosslinked alginate hydrogels so that photo-crosslink has attracted increasing attention in the preparation of alginate hydrogels [33,34]. Photo-crosslinked hydrogels are fabricated from a solution containing photoinitiators by the irradiation of ultraviolet or visible light [35,36]. Free-radical polymerization, which is usually used in microchannels to produce casting and ex-situ solidification, is generated through the photoinitiators in the crosslink process [12]. The concentration of the initiator has a strong influence on the rate and the uniformity of crosslinks dependent on the penetration depth and distribution of the light [37]. Meanwhile, some chemical photoinitiators (e.g., VA-086, VA-044, V-50, and Irgacure 1870) are cytotoxic to cells or bioactive molecules that are embedded in alginate hydrogels [35]. In order to reduce the amount of photoinitiator and its damage to cells or bioactive molecules without affecting the degree of crosslink, the active groups with photoinitiation, such as methacrylate (MA) are introduced into macromolecular chains [38,39]. For the hydrogels prepared by the photo-crosslinking process, most of their structures are mostly capsules, scaffolds, and thin films, meanwhile the photo-crosslinked hydrogels with fibrous structure are relatively less [6,33,34,35]. In the preparation of photo-crosslinked microfibers, the central solution can be solidified rapidly under UV irradiation, and the solidified polymer will affect the extrusion of the solution to a certain extent. So that a sheath solution with the viscosity the same as the central solution is needed to ensure the smooth extrusion of the central solution. Therefore, photo-crosslinking has limitations in the preparation of long hydrogel microfibers with continuous structure [17]. On the basis of calcium ionic crosslinking, adding photo-crosslinking can form hydrogels with more compact macromolecular chains entanglement and avoids the destruction of ionic crosslinked microfibers in physiological solution [40]. At the same time, it avoids the disadvantage of the photo-crosslinking process that the hydrogel microfibers with continuous structure can not be fabricated without lubrication solution. Therefore, dual crosslinking combines the advantages of ionic crosslinking and photo-crosslinking.

Spencer prepared the dual crosslinked methacrylated alginate microspheres. This microsphere has a more compact crosslinking structure than the single crosslinking process, which is conducive to the release of drugs embedded in it [40]. Jennifer used the MFF system to prepare double crosslinked alginate microspheres and researched its application in mesenchymal stem cell encapsulation [41]. The purpose of this study is to prepare dual crosslinked microfibers based on methacrylated alginate (Alg-MA) using the MFF system for the first time and explore its application in cell biology. Microfibrous hydrogels were prepared by calcium ionic crosslink and then the photo-crosslinking was used to improve the stability of the structure and mechanical properties of the hydrogel microfibers. The influence of different process parameters, including solution concentration, UV irradiation time on the structure and performance of the dual crosslinked hydrogel microfibers were investigated. The distribution and viability of cells encapsulated in the microfibers were examined in vitro. The hydrogel microfibers were fabricated into the hydrated woven network with stable mechanical properties and porous structure to explore the growth and proliferation of cells in three-dimensional (3D) fabric-like constructs through various pathways.

## 2. Results and Discussion

### 2.1. Characterization of Alg-MA and Dual Crosslinked Alg-MA Hydrogel Microfibers

#### 2.1.1. Synthesis of Alg-MA and Characterization

**Figure 1:**
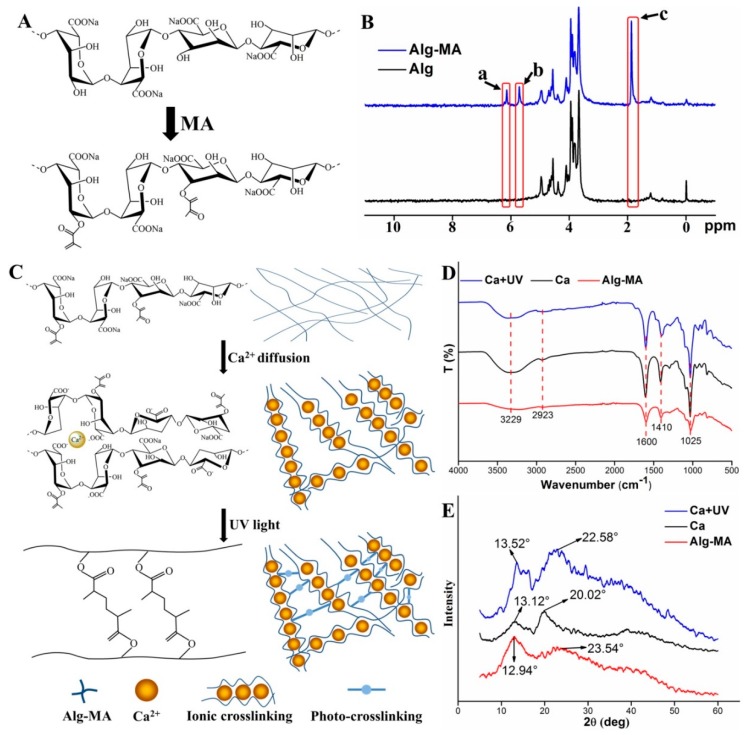
Chemical structure and characterization of synthesized methacrylated alginate (Alg-MA) and dual crosslinked hydrogel microfibers. (**A**) Schematic illustration of the synthesis of Alg-MA. (**B**) ^1^H-NMR spectra of alginate and Alg-MA. (**C**) Schematic illustration of chemical structure of dual crosslinked Alg-MA hydrogel microfibers. (**D**) FTIR spectra and (**E**) X-ray spectra of Alg-MA, ionic crosslinked and dual crosslinked Alg-MA hydrogel microfibers.

The introduction of methacrylate groups improves the photo-crosslinking performance of alginate at low concentration of photoinitiator [35]. As shown in Figure 1A, methacrylated alginate was formed by substituting part of hydroxyl groups of sodium alginate monomer with MA groups. The methacrylate modification of alginate was confirmed by the ^1^H-NMR spectra shown in Figure 1B. Compared with the ^1^H-NMR spectra of sodium alginate, peak a, b, and c represented the methacrylate groups in the modified alginate. The peaks a and b indicated the methylene proton peaks, and the methyl proton peak of the methacrylate groups was illustrated as peak c. The degree of methacrylation (DOM) of Alg-MA calculated from the ^1^H-NMR spectra was 61.31%. For polymers modified by methacrylate groups, the methacrylation efficiency results in changes of the crosslinking degree, structure, and properties of ionic crosslinking networks and photo-crosslinking networks. Moderate DOM is conducive to optimizing the dual crosslinking networks [40,42].

#### 2.1.2. Effects of Crosslinking on the Alg-MA Macromolecules Structure

As shown in Figure 1C, Ca^2+^ ions diffused into the Alg-MA solution at the top of the co-axial nozzle and replaced Na^+^ ions to form Ca ionic crosslinking. Ca^2+^ ions bonded with G group residues and formed an “egg-box” structure, which increased the stereoscopic properties of Alg-MA linear macromolecular chains. The ionic crosslinking occurs to the adjacent carboxyl side groups while the photo-crosslinking takes place at the hydroxyl groups, thus the dual crosslinking is obtained [40]. When the ionic crosslinked hydrogel microfibers were exposed to the UV light, under the action of photoinitiators, the photo-crosslinking between methacrylate groups improved the tight binding between alginate macromolecule chains, forming dual crosslinked Alg-MA hydrogel microfibers with more stable structure. Changes in the chemical structure of Alg-MA during crosslinking reaction were characterized by FTIR and XRD spectra (Figure 1D,E). Since no new functional groups were introduced and generated, no displacement occurred in the positions of functional groups in the FTIR spectra of Alg-MA. Meanwhile, the presence of moisture in Alg-MA hydrogel increased the absorption intensity of hydroxyl group at 3229 cm^−1^ after crosslinking. The bonding and entanglement between macromolecular chains enhanced the crystal structure and increased the intensity of the diffraction peaks at 2θ = 13° and 2θ = 22°. The crystallinity of Alg-MA, Ca ionic crosslinked Alg-MA and dual crosslinked Alg-MA was calculated as 24.37%, 35.87%, and 43.29%, respectively.

#### 2.1.3. Rheological Behaviors of Alg-MA Solution

**Figure 2:**
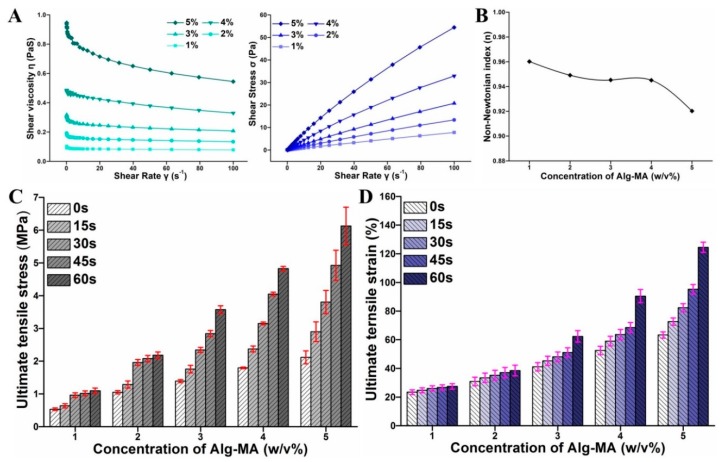
(**A**) The rheological behaviors of Alg-MA solution. (**B**) The non-Newtonian index variety curve. (**C**) The tensile stress of hydrogel microfibers with different Alg-MA concentrations. (**D**) The tensile strain of hydrogel microfibers with different Alg-MA concentrations.

The rheological properties of Alg-MA solution significantly affect its spinnability. In the experimental designs of the MFF system, the solution behavior under shear is a key factor to be considered [12]. Five different concentrations of Alg-MA (1%, 2%, 3%, 4%, and 5% (w/v)) were used in the fabrication of dual crosslinked Alg-MA hydrogel microfibers. As shown in Figure 2A, as the concentration increased from 1% (w/v) to 5% (w/v), the entanglement and the interaction among the Alg-MA macromolecular chains increased gradually, resulting in the viscosity increased from 0.15 to 1.01 Pa·S. With the increase of shear rate, the shear stress acting on the macromolecular chains increased, the entanglement and the interaction among the macromolecular chains were destroyed, which caused the decline of the viscosity. For a solution with low concentration, the entanglement between macromolecular chains was weak, so that the entanglement between macromolecular chains was destroyed by low shear stress and shear rate, leading to the rapid change of viscosity. For a solution with high concentration, the entanglement between macromolecular chains was strong, and low shear stress did not have a great influence on the interaction between macromolecular chains. With the increase of shear rate and shear stress, the interaction between macromolecular chains was gradually destroyed, which leads to the rapid decrease of shear viscosity at high shear rate and shear stress. Alginate solution is a pseudoplastic fluid, so its non-Newtonian index is below 1. For pseudoplastic fluid, the spinnability of solution declines with the decrease of non-Newtonian index [43,44]. Figure 2B illustrated that the spinnability decreased with the increase of the Alg-MA concentration. The spinnability of the solution changed inconspicuously with a concentration range from 1% (w/v) to 4% (w/v) and decreased when the solution concentration reached 5% (w/v). For Alg-MA solutions of different concentrations, when the Qc (flow rate of core solution) was less than 680 μL/min s^−1^, continuous metre-long microfibers were fabricated without destroying the entanglement of macromolecular chains and the solution properties.

### 2.2. Tensile Mechanical Properties

The tensile mechanical properties of fibers are mainly determined by the interaction strength between the macromolecular chains of fibers, which was composed of the strength of ionic junctions between Ca^2+^ ions and blocks and the covalent crosslinking strength of methacrylic groups. The amounts of Alg-MA macromolecular chains, the Ca^2+^ ions concentration, and the UV irradiation time were the main parameters affecting tensile mechanical properties. As shown in Figure 2C,D, the large number of macromolecular chains in Alg-MA solution with high concentration and the tight distance between macromolecular chains led to tense macromolecular chains entanglements. Since the high density of the “egg-box” junctions formed by Ca^2+^ ions and G blocks, the tensile stress and strain of ionic crosslinked microfibers increased significantly with the increase of Alg-MA concentration. This result was consistent with previous research that the increasing of sodium alginate improved the mechanical properties of gels [45,46]. Under UV irradiation, the formation of covalent crosslinking bonds between methacrylic groups of methacrylate increased the tensile strength and strain of dual crosslinked microfibers. With the increase of UV irradiation time, the degree and strength of photo-crosslinking enhanced, improving the tensile mechanical properties of dual crosslinked microfibers. With the increase of UV irradiation time, the tensile strength and strain of microfibers prepared with high concentration of methacrylic groups increased greater than those prepared with low concentration of methacrylic groups.

**Figure 3:**
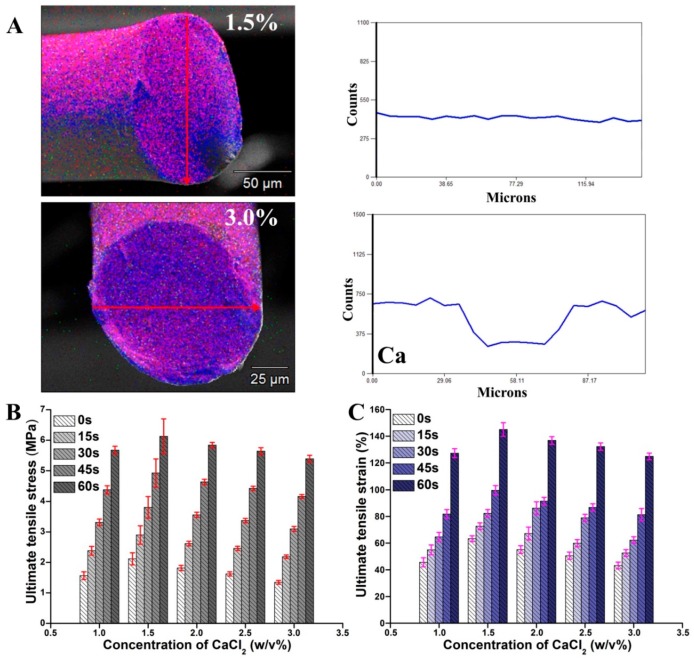
(**A**) Energy-dispersive X-ray (EDX) mapping images of the Alg-MA microfibers fabricated with 1% CaCl_2_ and3% CaCl_2_ solution. (**B**) The tensile stress of hydrogel microfibers with different CaCl_2_ concentrations. (**C**) The tensile strain of hydrogel microfibers with different CaCl_2_ concentrations.

The diffusion and distribution of Ca ions into the microfibers determined the degree and strength of Ca^2+^ ionic crosslinking. For Ca^2+^ ionic crosslinked alginate gel, the diffusion of Ca^2+^ ions in the gel network is determined by the concentration of Ca^2+^ ions, alginate solution ionic strength, and the porous structure in the gel network. As shown in Figure 3A, the Ca distributed in the microfibers fabricated by CaCl_2_ at 1.5% (w/v) and 3% (w/v) was visualized by the energy-dispersive X-ray (EDX) mapping. For low initial CaCl_2_ (1.5% (w/v)), the formed hydrogel structure was loose, Ca^2+^ ions diffused easily to the center of the hydrogel microfibers, forming a uniform Ca distribution. For high concentration CaCl_2_ solution, the gel structure is the main factor that influences calcium ions diffusion [47]. The high cation concentration alters the number of alginate strands participating in “egg-box” structure and formed a cooperative binding structure, which increases the tightness of the gel structure [46,48]. When a large number of calcium ions existed (3% (w/v)), the Alg-MA crosslinked rapidly and formed a tight hydrogel network at the top of the spinneret, which obstructed the diffusion of Ca^2+^ ions. The amount of Ca diffused to the center of microfibers was about 50% of that distributed on the surface.

Under the action Ca diffusion and cooperative binding, the gel strength of Ca^2+^ ionic crosslinked alginate hydrogel microfibers reaches the maximum at the CaCl_2_ concentration of 1.4% (w/v) [48]. As shown in Figure 3B,C, the tensile stress and strain of microfibers increased with the increase of CaCl_2_ concentration, reached the maximum at 1.5%, and then decreased gradually. With the increase of UV irradiation time, the tensile properties of the microfibers increased obviously, and the increase extents of the microfibers fabricated by CaCl_2_ with different concentrations were similar. The concentration of Ca^2+^ ions had no effect on the crosslinking between methacrylic groups.

### 2.3. Swelling Ratio Characteristics

**Figure 4:**
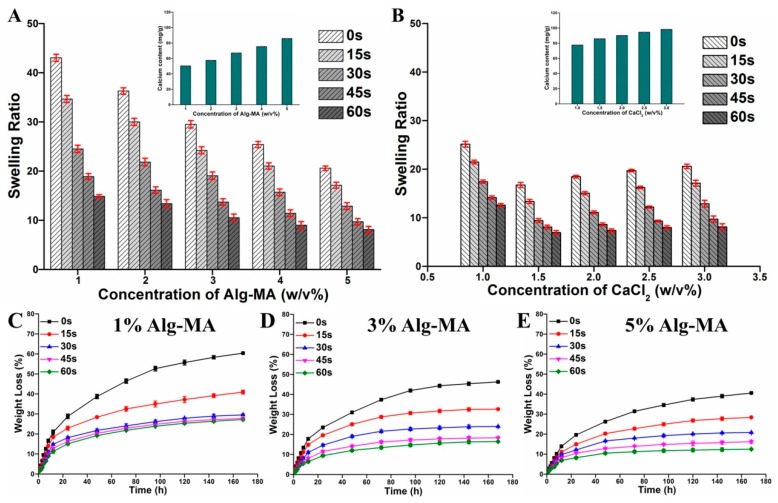
The stability characteristics of the Alg-MA hydrogel microfibers. (**A**) Swelling ratio of hydrogel microfibers with different Alg-MA concentrations. (**B**) Swelling ratio of hydrogel microfibers with different CaCl_2_ concentrations. Weight loss of hydrogel microfibers with (**C**) 1% (w/v) Alg-MA, (**D**) 3% (w/v) Alg-MA, and (**E**) 5% (w/v) Alg-MA.

The swelling degree of microfibers prepared with different Alg-MA concentrations, CaCl_2_ concentrations, and UV irradiation time was measured as described in the Materials and Methods section and illustrated in Figure 4A,B. The calcium ions chelated with M blocks residues can be replaced by sodium ions in solution changing Ca-Alg into soluble Na-Alg [42], which improves the swelling properties of alginate-based hydrogels. The crosslinking density and strength between macromolecular chains, Ca^2+^ ions content, the chemical bond strength between Ca^2+^ ions and blocks are the main factors affecting the swelling properties of gels. For ionic crosslinked microfibers, with the increase of Alg-MA concentration, the entanglement and crosslinking density between macromolecular chains and Ca^2+^ ions enhanced, which resulted in the decline of the swelling ratio of the microfibers, from 42 at 1% (w/v) Alg-MA to 23 at 5% (w/v) Alg-MA. The tight hydrogel networks formed by synergistic crosslinking between macromolecules reduced the swelling ratio of the microfiber from 25 at 1% (w/v) CaCl_2_ to 18 at 1.5% (w/v) CaCl_2_. The increasing of calcium ions in hydrogels improves the swelling capacity of microfibers [49,50,51]. The synergistic crosslinking between macromolecules disappeared for microfibers prepared by CaCl_2_ with concentrations above 2% (w/v). The Ca^2+^ ions content was the key factor affecting the swelling ratio of microfibers. The increase of initial CaCl_2_ concentration improved the Ca^2+^ ions content in the microfibers, and the swelling ratio of the microfibers gradually increased to 21 at 3.0% (w/v) CaCl_2_. With the increase of UV irradiation time, the density and intensity of crosslinking of macromolecular chains increased, resulting in more compact hydrogel networks resulting in a significant decrease in swelling ratio to 30 of the swelling ratio of ionic crosslinked microfibers at 60 s irradiation.

### 2.4. Weight Loss Characteristics

For hydrogels used in cell biology and biomedicine applications, it is very important to maintain the intact structure and stable performance in the cellular microenvironment [52]. However, the structure and properties of pure Ca^2+^ ionic crosslinked algnate materials are easily destroyed in physiological environments with chelation ions. In this study, the stability of the microfibers with 1%, 3%, and 5% (w/v) Alg-MA was characterized by the weight loss (Figure 4C–E). In simulated body fluid (SBF) solution, the hydrogel network structure of the ionic crosslinked microfibers disintegrated and the weight loss on 168 h of microfibers declined from 60% at 1% Alg-MA to 40% at 5% (w/v) Alg-MA. This confirmed that compact macromolecular entanglement improved the stability of network structure. The covalent crosslinking of methacrylate groups possesses high stability in cellular microenvironments. The addition of photo-crosslinking significantly reduced the weight loss of the microfibers, and the crosslinking density of the macromolecular chains and the tightness of the hydrogel network structure were enhanced by photo crosslinking with the increased UV irradiation time. The stability of the microfibers prepared at 60 s was three times greater than that of the ionic crosslinked microfibers. Meanwhile, the stability of microfibers prepared by high concentration Alg-MA was improved more by optical crosslinking than by low concentration Alg-MA.

### 2.5. Cell Distribution in the Dual Crosslinked Hydrogel Microfibers

**Figure 5:**
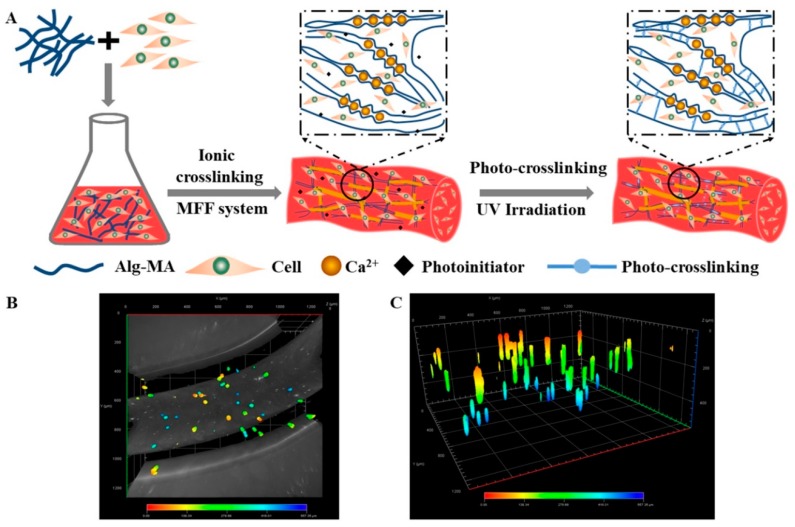
(**A**) The schematic diagram of fabrication of the cell-encapsulated Alg-MA hydrogel microfibers and cell distribution. The confocal images of cells distributed in the microfibers: (**B**) 2D distribution image and (**C**) 3D distribution image.

The hydrogel microfibers possess geometry similar to the internal networks of natural tissue so that hydrogel microfibers and their scaffolds can provide ideal biological matrices and constructs for tissue engineering [53]. The fabrication procedure of the cell-encapsulated Alg-MA hydrogel microfibers was illustrated in the schematic diagram (Figure 5A). The Alg-MA solution mixed with human umbilical vein endothelial cells (HUVECs) was extruded through the MFF system and HUVECs were encapsulated in the Ca^2+^ ionic crosslinked hydrogel network of the metre-long hydrogel microfibers. The photo-crosslinked hydrogel networks were generated by UV irradiation, providing more stable and permanent constructs for cells proliferation. Crosslinked Alg-MA macromolecular chains and cells interwove with each other to provide a stable spatial structure which allowed cells to adhere.

To obverse the distribution of the encapsulated cells in the microfibers with a cylindrical structure, the cell-encapsulated hydrogel microfibers were stained, and 3D scanned by confocal laser scanning microscope (CLSM). The fluorescence microscope images (Figure 5B,C) illustrated that HUVECs distributed randomly and homogeneously in the hydrogel microfibers and meanwhile the uniformity of cell distribution at the cross-section was better than that along the length direction of the microfiber. Compared with planar tissue engineering scaffolds, the hydrogel microfibers provided a 3D stereoscopic space for cells, which expanded the cell distribution.

### 2.6. Cell Proliferation in the Dual Crosslinked Hydrogel Microfibers

**Figure 6:**
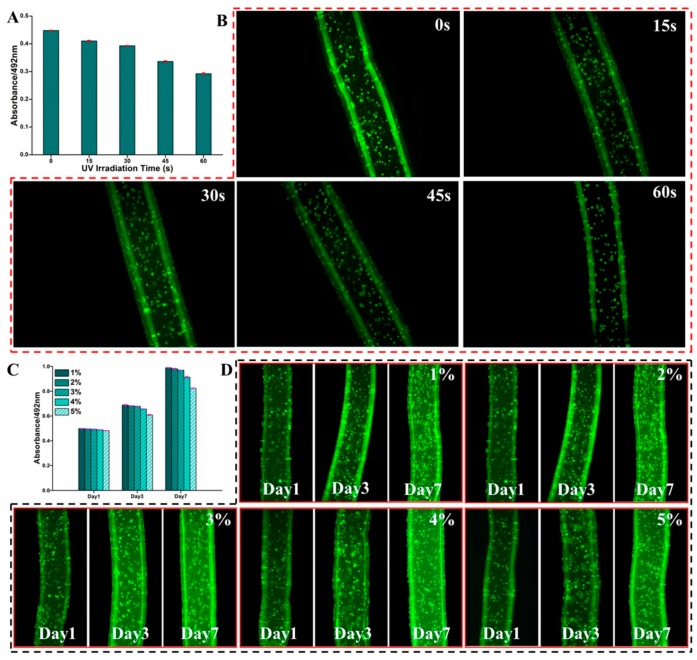
The viability and proliferation of cells in the cell-encapsulated Alg-MA hydrogel microfibers. (**A**) The cell viability and (**B**) the confocal images of the cells encapsulated in microfibers with different UV irradiation times. (**C**) The cell proliferation and (**D**) the confocal images of the cells encapsulated in microfibers with different Alg-MA concentrations.

Previous researches indicate that long-term UV exposure affects the viability of the encapsulated cells or even causes cell death [54,55]. Figure 6A compared the viability of cells encapsulated in microfibers with different UV irradiation times (15, 30, 45, and 60 s) and the ionic crosslinked microfibers were set as the control group. The cell viability was calculated by the absorbance ratio of the cells encapsulated in microfibers at 492 nm to that of the control cells. The viabilities of cells encapsulated in microfibers with UV irradiation time of 15 and 30 s were more than 85%, indicating that the encapsulated cells had good viability. As UV irradiation time increased to 45 and 60 s, the viability of cells encapsulated in microfibers declined rapidly to 75% and 65%, which proved that longer time of UV irradiation had a great effect on cell viability. Figure 6B illustrated the fluorescence micrographs of live encapsulated cells with different UV irradiation times. A large number of live cells were distributed in the microfibers with UV irradiation time below 30 s and the cells had intact morphology. When the UV irradiation time was above 45 s, the amount of live cells decreased significantly, and the morphological integrity of cells was slightly disrupted.

Microfibers fabricated by the MFF system have been demonstrated to provide excellent conditions for cell proliferation. Human fibroblast cells cultured for 7 days in alginate microfibers remain viable culture [30]. To explore the potential of the dual crosslinked hydrogel microfibers in cell biology, the cell proliferation assay was performed and is illustrated in Figure 6C. It was shown that the absorption of the encapsulated cells increased obviously with an extension of culture time, proving that the encapsulated cells maintained activity and the microfibers had excellent cytocompatibility. Meanwhile, the proliferation of cells encapsulated in microfibers prepared by Alg-MA with concentration below 3% (w/v) was better than that prepared by Alg-MA with concentration above 4% (w/v). The stained cells were observed by CLSM to character the morphology and spreading of the cells encapsulated in the microfibers with different concentrations (Figure 6D). After 1 day of culture, the encapsulated cells did not proliferate and spread noticeably and the cells with rounded shapes distributed within the microfibers in a dispersed pattern. With the growth and proliferation of cells, the amount of cells in the microfibers increased significantly and the cells gradually spread out in different directions. The cell’s morphology gradually changed to spindle shapes and the cell’s distribution pattern changed from single dispersed distribution to cell clusters distribution pattern. Both the proliferation amount and spread scale of the cells encapsulated in microfibers prepared by Alg-MA with concentration below 3% (w/v) were greater than those prepared by Alg-MA with concentration above 4% (w/v), confirming that the hydrogel microfibers prepared at low Alg-MA concentrations were more conducive to cell growth, proliferation, and spreading. For the hydrogel microfibers with high concentration Alg-MA, the density and stiffness of the hydrogel network formed between macromolecular chains were high. The scaffolds with similar mechanical properties have been confirmed to be detrimental to cell proliferation, spreading, and migration [54,56].

### 2.7. Assemblies of the Cell-encapsulated Dual Crosslinked Hydrogel Microfibers

**Figure 7:**
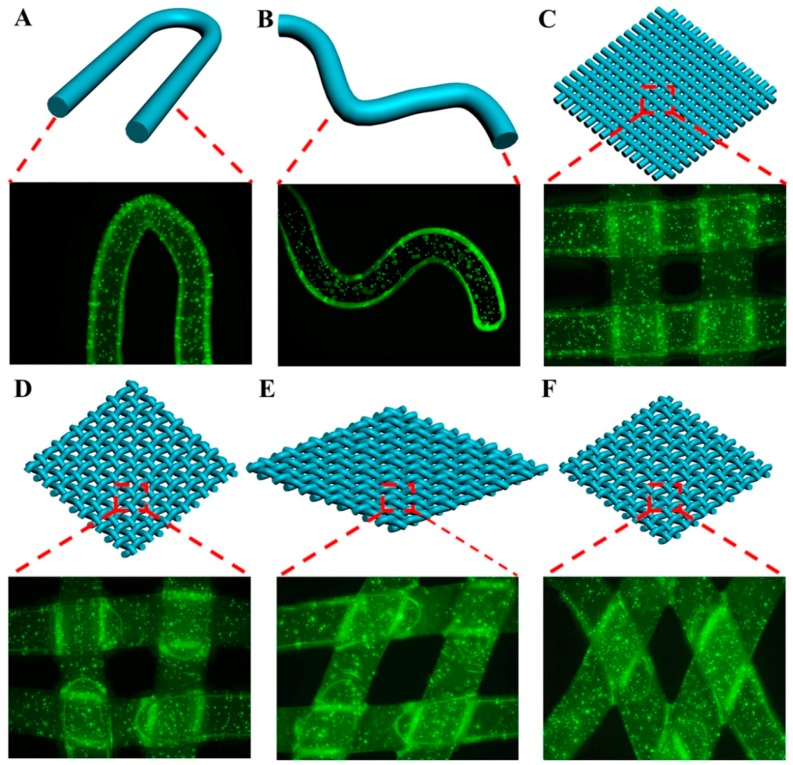
Schematic illustrations and confocal laser scanning microscope (CLSM) images of (**A**,**B**) the cell-encapsulated microfibers with flexural structure; (**C**–**E**) textile assembly constructs consisting of crisscross woven cell-encapsulated microfibers; (**F**) layer-by-layer construct stacked with the cell-encapsulated microfibers.

As a textile assembly, weaving possesses a large-area structure with internal hierarchy [12] that has been used as tissue engineering biomaterials [17]. The “tissue-weaving” can produce cell-encapsulated microfibers with hierarchy structure into cell-encapsulated textiles with a second-generation hierarchy structure and becomes an emerging strategy to prepare next-generation regenerative medicines. The weaving provided microfibers a stable framework structure with excellent properties while the hydrogel microfibers protected the viability of cells [57]. As shown in Figure 7A,B, the thin Alg-MA microfibers possessed excellent plasticity, flexibility, and high tensile strain, which ensured that the microfibers maintained the stability of structure and performance in states of bending and crook, and provided excellent growth conditions for the cells. To investigate the potential of the flexible Alg-MA microfibers to produce “biological-textiles” with 3D tissue structure, the microfibers were crisscross weaved to prepare a framework with 3D gridding constructs (Figure 7C–E). Fabric-like scaffolds with different spatial structures and properties were obtained by changing the angle between crossed microfibers. The microfibers were also cross stacked to create a 3D layer-by-layer architecture (Figure 7F). From the confocal diagrams of the stained cells in the fabric-like scaffolds cultured for 7 days, the 3D constructs of the textile assembly maintained their stable structures, the microfiber structure in the textile assembly was well protected, providing beneficial conditions for cell growth, proliferation, and spread. This provided support for the applications of the microfibers in fabrication of the fabric-like tissue constructs which had high potential as the next-generation cell-encapsulated scaffolds in tissue engineering and regenerative medicine.

## 3. Materials and Methods

### 3.1. Materials

Alginic acid sodium salt (alginate from brown algae, low viscosity), methacrylic anhydride (MA), water-soluble photoinitiator Irgacure 2959 (2-hydroxyl-4′-(2-hydroxyethoxy)-2-methylpropiophenone), and Deuterium oxide (D_2_O) were purchased from Sigma-Aldrich (St. Louis, Mo, USA). Sodium hydroxide (NaOH), calcium chloride (CaCl_2_), and dimethyl sulfoxide (DMSO) were obtained from Aladdin (Shanghai, China). Reagents used in cell culture and MTT assay were purchased from Gibco (Thermo Fisher Scientific Co., Waltham, MA, USA). Calcein-AM was procured from KeyGEN BioTECH Co. (Nanjing, Jiangsu, China). All the reagents used to prepare SBF solution [50] were purchased from Sigma-Aldrich (St. Louis, Mo, USA). The water purified by a Milli-Q water purification system (Millipore, Bedford, MA) was used in all the experiments and the resistivity higher than 18.2 MΩ.

### 3.2. Alg-MA Synthesis and Characterization

Alg-MA was prepared based on the synthesis methods of previous studies [12]. Briefly, sodium alginate was dissolved in Milli-Q water and stirred for 4 h to prepare 2.0% (w/v) alginate solution. The alginate solution was adjusted to pH 8 by adding 0.5 M NaOH solution. Excess methacrylic anhydride (20-fold) was dropped into the alginate solution and the mixture was stirred at 4 °C for 24 h. During this process, using 0.5 M NaOH solution to keep the pH of synthesis system at 8.0. Then, the mixture was dialyzed in Milli-Q water for 48 h to remove excess MA and followed by lyophilization to generate the final modified alginate. The methacrylated alginate (6.5 mg) was dissolved in deuterium oxide (1 mL) and measured by the ^1^H NMR spectrum (AVANCE III 500 MHz NMR spectrometer, Brucker, San Jose, CA, USA). The DOM was calculated by the ratio of the methyl protons to the methylene protons of methacrylate.

### 3.3. Preparation of the Dual Crosslinked Alg-MA Hydrogel Microfibers

Alg-MA was dissolved in Milli-Q water with 0.05% (w/v) Irgacure 2959 and stirred for 8 h to prepare solution with concentrations of 1%, 2%, 3%, 4%, 5% (w/v). The rheological behaviors (shear viscosity, shear stress, non-Newtonian index) of Alg-MA solution were measured by ARES rheometers (TA Instruments Co., New Castle, DE, USA) to evaluate its spinnability. The diameter of the rotor was 20 mm, and the speed of the rotor increased gradually from 0 r/s to 100 r/s at the rate of 5 r/s.

**Figure 8:**
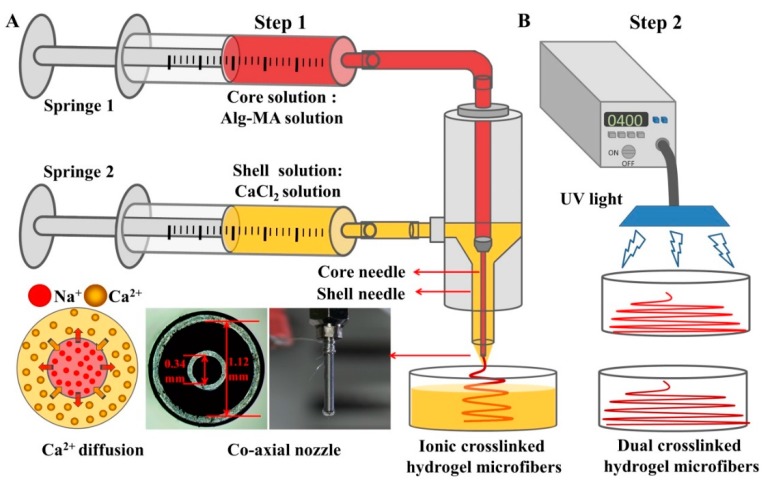
Schematic illustration for the fabrication of the dual crosslinked Alg-MA hydrogel microfibers. (**A**) Ionic crosslinked Alg-MA hydrogel microfibers fabricated by the Microfluidic Fabrication (MFF) system. (**B**) Photo-crosslinking caused by UV light.

As shown in Figure 8, the dual crosslinked hydrogel microfibers were fabricated by two-steps. Firstly, the ionic crosslinked Alg-MA (Ca-Alg-MA) hydrogel microfibers were fabricated by the MFF system which consists of two digital syringe pumps (RSP01-BDG, Ristron Electronic Technology Co., Ltd., Jiaxing, Zhejiang, China) and a spinning nozzle assembled by two metal needles with a co-axial arrangement (Changsha Nano Apparatus Technology Co., Ltd., Changsha, Hunan, China). The Alg-MA solution and CaCl_2_ solution were extruded by core and shell needles respectively and contacted at the top of the co-axial nozzle. With the continuous extrusion of Alg-MA and ions exchange reaction, metre-long ionic crosslinked Alg-MA hydrogel microfibers were fabricated. Then, the ionic crosslinked microfibers were exposed to UV light (360–480 nm, 6.9 mWcm^‒2^ power) for the prescribed time. Under the action of photoinitiator, covalent crosslinking was added, forming dual crosslinked hydrogel microfibers. The redundant CaCl_2_ and photoinitiator were removed by washing by Milli-Q water 3 times.

The functional groups were examined through Nicolet 6700 FT-IR spectroscopy (Thermo Fisher Scientific USA) with attenuated total reflection (ATR) mode in the range of 500–4000 cm^−1^. The effect of crosslinking on the aggregation structure of Alg-MA macromolecules was analyzed with the X-ray spectrum measured by a D/max RB X-ray diffractometer (Rigaku Co., Tokyo, Japan) with Nickel-filtered Cu kα radiation in the 2θ range of 0°–60°.

### 3.4. Tensile Mechanical Testing

**Figure 9:**
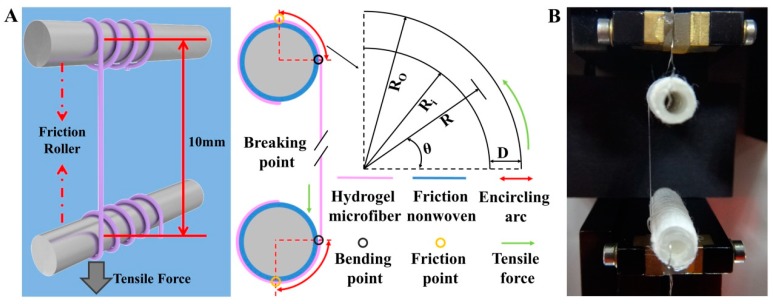
The tensile properties test system of hydrogel microfiber: (**A**) the schematic diagram. D: Diameter of the microfiber; R and θ were the radius and the angle of any point of the microfiber in the bending region, respectively. R_i_ and R_0_ were the inner and the outer radius of the microfiber in the bending region, respectively. (**B**) the instrument used in the tensile experiments.

The tensile properties of fiber were tested by a single fiber strength tester and the fiber was clamped in the chucks. But the shear force produced by the chucks could cut off the hydrogel microfiber. Therefore, the single hydrogel microfiber was twined around a friction roller coated by nonwoven fabric with fluffy and rough surface to avoid direct contact with the clamp that might damage the microfibers (Figure 9). The tensile properties of the hydrogel microfiber were tested by XQ-1 Electronic Single Fiber Strength Tester (Laizhou Electron Instrument Co., Laizhou, Shandong, China). The gauge length and the constant deformation speed were set as 10 mm and 10 mm/min, respectively.

### 3.5. Swelling Testing

The prepared microfibers were immersed in 10 mL PBS at 37 °C. After 24 h swelling, the microfibers were removed from PBS solution and rinsed 3 times with Milli-Q water. After removing the excess solution on the microfibers surface with filter paper, the swollen microfibers were weighed (W_s_). The dry microfibers after lyophilization were weighed (W_d_). The degree of swelling (DOS) was calculated according to the formula: DOS = (W_s_ ‒ W_d_)/ W_d_.

### 3.6. Weight Loss Testing

The prepared microfibers were placed in a centrifugal tube containing 10 mL SBF refreshed every day and incubated at 37 °C. After desired time (0.5, 1, 2, 3, 5, and 7 days), the degraded microfibers were removed from SBF and washed with Milli-Q water 3 times. The microfibers were lyophilized and weighed. The weight loss (WL) was calculated according to Equation (1):

WL = (W_0_ ‒ W_1_)/W_0_ × 100% (1)

Here, W_0_ and W_1_ were the dry weight of microfibers before and after degradation assay, respectively.

### 3.7. Cell Culture and Preparation of Cell-Encapsulated Dual Crosslinked Hydrogel Microfibers

Human umbilical vein endothelial cells (HUVECs) for encapsulation were obtained from Cell Bank of the Chinese Academy of Science, Shanghai, China) and cultured in medium consisting of 90 v/v% high glucose DMEM, 10 v/v% fetal bovine serum (FBS), and 1 v/v% penicillin/streptomycin at 37 °C and 5% CO_2_.

Alg-MA was dissolved in DMEM with 0.05% (w/v) Irgacure 2959 to prepare Alg-MA-DMEM solution. The Alg-MA-DMEM solution was sterile-filtered through a 0.22 μm Filter Unit, (Merck Millipore Ltd., Cork, Ireland). The trypsinized HUVECs were suspended in the Alg-MA-DMEM solution at a concentration of 3 × 10^5^ cells/mL. CaCl_2_-DMEM solution was prepared and sterile-filtered. The cell-encapsulated dual crosslinked Alg-MA hydrogel microfibers were fabricated as described in Section 3.3 with sterilized equipment in a sterile chamber. The prepared cell-encapsulated microfibers were washed with DMEM three times and cultured in medium at 37 °C and 5% CO_2_. HUVECs in microfibers were stained by calcein-AM (KeyGEN BioTECH Co., Nanjing, Jiangsu, China) and visualized by confocal laser scanning microscope (CLSM LSM 700, Carl Zeiss, Oberkochen Germany) to investigate the cell distribution.

### 3.8. Cell Viability and Proliferation in Cell-encapsulated Dual Crosslinked Hydrogel Microfibers

The viability and proliferation of HUVECs were measured by MTT assay and observed by cell staining assay. After the certain period of culture, the medium was removed, and the cell-encapsulated microfibers were washed with PBS for 3 times. Calcein-AM working solution was added to stain the live cells. The cell-encapsulated microfibers were incubated with staining solution for 30 min and observed by an inverted fluorescence microscope (IX71, Olympus, Tokyo, Japan). After the certain period of culture, the medium was replaced by 80 μL 0.5% (w/v) MTT-PBS solution and 720 μL DMEM. After 4 h of incubation, MTT solution was replaced by 800 μL dimethyl sulfoxide (DMSO) and oscillated on concentrator at 37 °C for 30 min. The absorbance value (O.D. Values) at 492 nm was measured by a microplate reader (Multiskan MK3, Thermo, Waltham, MA, USA).

### 3.9. Assembly of Cell-encapsulated Dual Crosslinked Hydrogel Microfibers

The prepared cell-encapsulated dual crosslinked hydrogel microfibers were assembled into scaffolds with fabric-like tissue constructs including crisscross woven with different arrangements and layer-by-layer structure by manual manipulation. After 7 days culture, the assembled scaffolds were stained with calcium AM and observed with an inverted fluorescence microscope.

## 4. Conclusions

In this research, Alg-MA was synthetized and produced into continuous Ca^2+^ ionic crosslinked microfibers with cylindrical structure by the MFF system. Under UV irradiation, covalent crosslinking formed between methacrylic groups, improving the tensile mechanical properties. Compared with the Ca^2+^ ionic crosslinked hydrogel microfibers, the swelling ratio of the dual crosslinked hydrogel microfibers in PBS decreased by 60%, and the weight loss on day 7 of the dual crosslinked hydrogel microfibers in SBF was only 25% of that of Ca^2+^ ionic crosslinked hydrogel microfibers. The dual crosslinked hydrogel network structure significantly improved the stability of the hydrogel microfibers in physiological solution. Cell-encapsulated microfibers with uniform cellular distribution were fabricated by adding specific cells into Alg-MA solution before extrusion. The stable stereoscopic structure provided admirable conditions for cell growth and proliferation. The cell-encapsulated microfibers were weaved to fabric-like frameworks to produce cell- encapsulated scaffolds with functional 3D tissue structures and better mechanical properties. The status of cells cultured in these microfiber scaffolds indicated that these scaffolds had great potential in the production of next-generation scaffolds for tissue engineering and regenerative medicine. We believe that the dual-cross-link method could be used to produce stable cell-encapsulated microfibers-based scaffolds with various 3D structures which had wide applications in tissue engineering.
